# Characteristics and Treatment Preferences of People with Symptoms of Posttraumatic Stress Disorder: An Internet Survey

**DOI:** 10.1371/journal.pone.0021864

**Published:** 2011-07-19

**Authors:** Jay Spence, Nickolai Titov, Karen Solley, Blake F. Dear, Luke Johnston, Bethany Wootton, Alice Kemp, Gavin Andrews, Judy Zou, Carolyn Lorian, Isabella Choi

**Affiliations:** 1 Clinical Research Unit for Anxiety and Depression (CRUfAD), St Vincent's Hospital, Sydney, Australia; 2 School of Psychiatry, University of New South Wales, Sydney, Australia; The University of Queensland, Australia

## Abstract

**Background:**

Although Posttraumatic Stress Disorder (PTSD) is a severe and disabling anxiety disorder, relatively few people with this condition access evidence-based care. Barriers to treatment are multiple and complex, but the emerging field of Internet therapy for PTSD may improve access to evidence-based treatment. However, little is known about the characteristics of people with PTSD who seek online treatment, or whether they perceive internet treatment as an acceptable treatment option.

**Methodology:**

An online survey was used to collect information about the demographic and symptom characteristics of individuals with elevated levels of PTSD symptoms, and this was compared to data from corresponding sample from a national survey. Previous treatment experiences, perceived barriers to treatment and treatment preferences for Internet therapy and face-to-face treatment were also compared.

**Principal Findings:**

High levels of PTSD symptoms were reported by survey respondents. Psychological distress and disability was greater than reported by individuals with PTSD from a national survey. Half of the sample reported not having received treatment for PTSD; however, 88% of those who reported receiving treatment stated they received an evidence-based treatment. Primary barriers to treatment included cost, poor awareness of service availability, lack of prior treatment response and not perceiving personal distress as severe enough to warrant treatment. Most survey respondents indicated they were willing to try Internet treatment for PTSD.

**Conclusions:**

The Internet sample was symptomatically severe and multiple barriers existed to treatment. Internet therapy is an acceptable option for the treatment of PTSD in an internet sample.

## Introduction

Posttraumatic Stress Disorder (PTSD) is one of the most prevalent of the anxiety disorders and is estimated to affect up to 6% of the adult population in a 12-month period [1]. PTSD commonly co-occurs with other psychiatric disorders, and is associated with considerable disability [2], [3], [4]. However, while effective psychological treatments exist for PTSD [5], [6], [7] only a minority seek treatment.

Barriers to treatment experienced by people with PTSD are similar to those experienced by people with other anxiety disorders and include the direct and indirect costs of treatment, personal beliefs about the efficacy or lack of efficacy of treatment, and shame about their symptoms [8], [9], [10]. One recent strategy that has potential for reducing barriers to evidence-based treatment is Internet-based therapy, particularly internet-delivered cognitive behavioural therapy (iCBT). iCBT involves the administration of the same techniques and materials usually delivered in face-to-face treatment, but is instead administered over the internet and may include regular communication with a healthcare worker via email, telephone, or other electronic media [11]. Recent meta-analyses indicate that Internet therapy is both an efficacious and effective treatment for depression and several anxiety disorders, and produces results comparable to face-to-face treatment [12], [13], [14].

Several studies have explored the efficacy of treating PTSD symptoms via the Internet [15], [16], [17], [18], [19], [20]. These studies have utilized evidence-based treatment components for PTSD symptoms such as in vivo and imaginal exposure, cognitive restructuring and de-arousal strategies. These programs have shown promise in reducing PTSD symptoms in military personnel, university students, and community samples with between-group effect sizes (Cohen's d) of between 0.4 and 1.4 reported on measures of PTSD symptoms [15], [16], [21]. These preliminary results indicate the potential of Internet therapy as a treatment option for PTSD, which may also improve access to treatment. While Internet therapy programs for PTSD appear promising, little is known about the characteristics of individuals with PTSD seeking treatment online, or whether consumers with PTSD would use such interventions. Furthermore, it has been estimated that 23–40% of individuals with PTSD seek help from a professional [22], [23], [24]; however, no research has specifically reported the uptake of evidence-based treatments for PTSD such as trauma-focused cognitive behavioral therapy or psychotropic medications. The findings from this study will help to determine whether online treatment seekers with symptoms of PTSD are representative of the general population with PTSD as well as the reasons why they are seeking Internet-based treatments.

The primary aim of the present study was to compare the characteristics of Australian adults with PTSD who completed an internet-based survey (Internet sample) with the characteristics of those identified in a national survey (national sample). Based on a similar study comparing characteristics of people with anxiety and depression [25], it was expected that the Internet sample would have more severe symptoms than those identified in the national sample. The secondary aims were to examine evidence-based service utilization and barriers to treatment in the Internet sample, and to examine the acceptability of Internet therapy to this group. It was expected that respondents to the Internet survey would rate Internet therapy as an acceptable potential treatment option and would report willingness to participate in such interventions.

## Methods

### Participants

The Internet sample (n = 244) was a convenience sample of respondents to an Internet survey (see below) about PTSD and trauma hosted on the website of a research clinic conducting research on Internet treatments for depression and anxiety (www.virtualclinic.org.au) between March 2010 and December 2010. The VirtualClinic is a joint venture between the University of New South Wales and St Vincent's Hospital, Sydney. There were no incentives for participation, participants were not required to be enrolled in a research trial or to log in to the site and individuals involved in research trials were not specifically encouraged to participate. Participants were clearly informed that participation in the survey would not influence their inclusion in any future trial.

The inclusion criteria were: 1) Resident of Australia; 2) between the age of 18 and 64 years; 3) completed the entire survey; 4) reported experiencing at least one traumatic event in their lifetime; 5) experienced a traumatic event occurring more than one month ago, and; 6) obtained a total score above 44 on the Post-Traumatic Stress Disorder Checklist – Civilian Version (PCL-C; [26]) with a score of 3 or more for each symptom cluster, considered a clinically significant cut-off consistent with a DSM-IV diagnosis of PTSD [27].

The national sample were identified from a sample of 8,841 Australian adults aged 16–85 years who were interviewed by trained interviewers from the Australian Bureau of Statistics in a large epidemiological national survey of common mental disorders (National Survey of Mental Health and Wellbeing; NSMHWB) [3]. Clinical diagnoses were made using the Composite International Diagnostic Interview, Version 3.0 (CIDI 3.0) [24]. Interviews were conducted face-to-face between August and December 2007 with a response rate of 60%. In order to identify the PTSD subsample from this survey the following inclusion criteria were applied: 1) Between the age of 18 and 64 years, and; 2) met DSM-IV diagnostic criteria for lifetime PTSD and clinically significant symptoms in the previous 12 months; however, PTSD was not necessarily their primary diagnosis. The data from the resultant sample of 400 individuals who met these inclusion criteria were analysed.

### Ethics and Survey Design

This study was approved by the Human Research Ethics Committees for the University of New South Wales and ratified by St Vincent's Hospital, Sydney. All participants provided written informed consent.

The survey comprised of 76 items broken into four sections addressing: 1) Demographics, psychological distress and disability (61 items); 2) PTSD symptoms and trauma experiences (6 items); 3) treatment history and barriers to treatment (4 items), and; 4) acceptability of internet treatment (5 items). See Appendix S1 for treatment definitions used in the survey. The questions and functionality of the survey were pilot tested on two occasions, and questions were subsequently modified to improve comprehension. No formal analyses of internal consistency or validity were conducted.

Demographic questions included questions about age, gender, marital status, education and employment, and was based on similar questions asked in the NSMHWB [3]. Information about psychological distress, disability, PTSD symptoms and trauma experiences was obtained by the administration of several psychometric measures (see below), two of which were also administered in the NSMHWB. Treatment history was assessed by asking if the respondent had ever been treated for PTSD. Respondents who reported receiving treatment were asked to indicate the types of treatment received, with definitions for these reflecting those used in the NSMHWB. Barriers to accessing treatment were assessed by asking respondents to indicate their main reasons were for not getting therapy or not seeking further therapy for PTSD according to 17 response options including one open response. Response options were collated from previous surveys assessing barriers to treatment [9], [10], [23], [28], [29], [30]. Respondents were also asked whether there was a mental health professional that was trained to treat PTSD who they could see for face-to-face treatment.

Acceptability of internet treatment was assessed by asking Internet respondents if they would try Internet-therapy for PTSD. Respondents were also asked about their preference for face-to-face therapy compared with Internet-based therapy on a 7-point Likert scale ranging from *Very definitely prefer face-to-face to Very definitely prefer Internet treatment* plus an option for those *not seeking treatment*.

### Measures

Demographic information provided by both groups included country of residence, age, gender, marital status, education and employment, using the same questions as used in the NSMHWB.

#### Kessler 10 Item (K-10) [29]

Psychological distress was also measured in both samples using the K-10 [29], which is a 10-item self-report measure of psychological distress. The scale has high internal consistency with a Cronbach alpha of 0.92 [31]. The internal consistency in this study was .93. The K-10 has been shown to be sensitive to treatment change.

#### World Health Organization Disability Assessment Schedule, 2^nd^ Edition (WHODAS-II) [30]

Disability was measured in both samples using the WHODAS-II [30], which is a brief measure of general functioning and the degree of interference due to symptoms. The WHODAS-II has a reported internal consistency of 0.86 and high test-retest reliability [32]. The internal consistency in this study was .92.

#### Post-traumatic Stress Disorder Checklist – Civilian Version (PCL-C) [22]

PTSD symptoms were measured in the IS but not the national sample, using the PCL-C [22], a reliable and valid measure of PTSD symptomatology based on the DSM-IV criteria for PTSD [33]. The internal consistency of the PCL-C in this study was .91. Participants rated the frequency of 17 PTSD symptoms within the past month from 1 (“not at all”) to 5 (“extreme”). For screening purposes, the PCL-C was scored according to recommendations shown to result in a sensitivity of .90 and a sensitivity of .95 [27]. Inclusion requirements necessitated a score of 4 or 5 on items 1, 2, 9, 10, 12, and 15; and a score of 3 or more for the remaining 11 items.

#### Life Events Checklist (LEC) [33]

Information on trauma history of the Internet sample was collected using the LEC [33]. The LEC provides a list of traumatic events and assesses the occurrence rates of common Criterion A1 (life-threatening) traumas according to the DSM-IV.

### Statistical Methods

Categorical data was analysed using chi-square tests, while mean differences in age and symptom severity were assessed using one-way *t*-tests. The results are reported in sections examining: 1) Demographic and symptom comparisons between the Internet and national samples; 2) acceptability of Internet therapy for PTSD as perceived by the Internet sample, and; 3) the treatment experiences and perceived barriers to treatment of the Internet sample. Analyses were performed using the Statistical Package for Social Sciences (SPSS) version 18.0 for Windows.

## Results

Of the 308 respondents who completed the Internet survey 2 were excluded because they were not Australian residents, 2 for being younger than 18 years of age, 29 for not having experienced a traumatic event, 4 because their major trauma had occurred less than a month ago, and 27 for having a total score below the clinical cut-off on the PCL-C. The data for 244 remaining participants was analysed. The data structure met the required assumptions for statistical analysis using parametric techniques.

### 1. Symptom Characteristics

#### Demographic Characteristics

Demographic characteristics of the two samples are displayed in Table 1. The Internet sample had a higher level of educational qualifications than the national sample (χ^2^ = 29.4, p<0.001); however, there were no significant differences between the samples in age, gender, marital status or employment status.

**Table 1:** Demographic characteristics and symptom severity of the Internet Survey (IS) and the National Survey (NS) samples.

Category	Subcategory	IS (N)	IS Mean (SD) / Percent	NS (N)	NS Mean (SD) / Percent	Test Statistic	p-Value
***Mean Age (SD) / Age in Categories (%)***		244	41.8 (12.2)	400	41.6 (15.2)	*t* = −1.5	.89
	18–24 years	17	7%	16			
	25–24 years	58	24%	19			
	35–44 years	65	27%	25			
	45–54 years	61	25%	18			
	55–64 years	39	16%	14			
	65+ years	4	2%	8			
***Gender (% male)***		244	22.5%	400	26.5%	*X*2 = 1.07	.30
***Marital Status (%)***		244		400		*X*2 = 1.51	.47
	Single / Never Married	87	35.7%		37.3%		
	Married / Defacto	93	38.1%		33.5%		
	Separated / Divorced / Widowed	64	26.2%		29.3%		
***Highest Educational Qualification (%)***		244		400		*X*2 = 29.37	<.0005
	No qualification / High School	57	23.4%		40.0%		
	Vocational qualification / other certificate	46	18.9%		23.5%		
	Diploma / Degree or above		57.8%		36.5%		
***Employment Status***		244		400		*X*2 = 3.26	.06
	Employed full-time or part-time	131	53.7%	61.3			
	Unemployed / Not working	113	46.3%	38.8			
***Psychological Distress (K-10)***		244	32.4 (7.7)	400	20.7 (7.9)	*t* = −18.3	<.0005
***Disability (WHODAS-II)***		244	32.1 (9.5)	400	19.9 (7.8)	*t* = −17.5	<.0005

K-10: Kessler 10-Item Scale; WHODAS-II: World Health Organisation Disability Assessment Schedule – Second Edition.

#### Psychological Distress and Disability

The Internet sample reported significantly higher K-10 and WHODAS-II scores (*t* (642)  =  −18.3, p =  <.0001 and t (642)  =  −17.5, p =  <.0001, respectively) than the national sample.

### 2. Ptsd Symptoms and Trauma Experiences

#### PTSD Symptoms

The Internet sample had a mean score of 60.4 (SD = 13.1, range: 44–85) on the PCL-C.

#### Frequency and Type of Trauma

The Internet sample reported experiencing a mean of 5.2 (SD = 2.6) types of trauma in their lifetime according to the LEC, with 173 (71%) respondents endorsing 4 or more types of trauma experience. The five most frequently endorsed types of trauma (number and percentage endorsing experiencing that trauma type) were *physical assault* (*n* = 165, 70%), *other unwanted or uncomfortable sexual experience* (*n* = 152, 64%), *any other stressful event or experience* (*n* = 147, 62%), *sexual assault* (*n* = 134, 57%), and *sudden unexpected death of someone close* (*n* = 102, 43%). Detail of trauma type and prevalence is reported in Table 2. Ninety percent of the Internet sample reported experiencing 2 or more distinct types of traumas, 80% reported experiencing 3 or more, 71% reported experiencing 4, 57% reported experiencing 5 or more, 41% reported experiencing 6 or more, and 29% reported experiencing 7 or more.

**Table 2:** Trauma type experiences reported by the Internet sample (*n* = 244).

Trauma Type	(n)	%
Physical assault	171	70%
Other sexual	156	64%
Other	151	62%
Sexual assault	139	57%
Unexpected death	105	43%
Transportation	98	40%
Illness / Injury	83	34%
Weapon assault	78	32%
Severe suffering	51	21%
Accident	46	19%
Natural disaster	34	14%
Fire or explosion	31	13%
Violent death	27	11%
Captivity	26	11%
Caused injury	24	10%
Toxic exposure	20	8%
Combat or war	15	6%

### 3. Treatment History and Barriers to Treatment: Internet Sample

#### Prior Treatment of PTSD

One-hundred (41%) of the Internet sample reported *having received treatment for PTSD*, 24 (10%) were *unsure*, and 120 (49%) reported that they *had not received any treatment for PTSD*. The mean number of types of treatment received was 4 (SD = 2.4). Of those who indicated receiving treatment, eighty-one (81%) respondents reported receiving pharmacological treatments, seventy-eight (78%) reported receiving counselling and sixty-nine (69%) reported receiving cognitive behavioural therapy. Seventy-nine (79%) received CBT and medication, and eighty-eight (88%) received CBT or medication. Two people (1%) reported receiving CBT only, eight people (3%) reported receiving counselling only, and twelve people (5%) reported receiving medication only.

#### Availability

One hundred and seventeen (48%) of the Internet sample reported they were not sure whether there was a mental health professional that was trained to treat PTSD in their local area. Ninety (37%) reported that a trained professional was available, and 37 (15%) said that none were available.

#### Barriers to therapy

Data about the perceived barriers to treatment are included in Table 3. The barriers reported by more than 20% of the Internet sample for *not getting therapy or not seeking further therapy for PTSD* were lack of money (*n* = 105, 43%), lack of treatment response from previous therapy (*n* = 70, 30%), not wanting to take medication (*n* = 58, 24%), and not perceiving their difficulties as severe enough to warrant therapy (*n* = 51, 21%).

**Table 3:** Barriers to therapy/further therapy for the Internet sample (*n* = 244).

Reason for not getting therapy /not seeking further therapy for PTSD	(n)	%
Lack of money	105	43%
Previous therapy didn't work	72	30%
I didn't want to take medication	58	24%
I didn't think my problem was severe enough	51	21%
I didn't want to be seen as weak	47	19%
I thought therapy would be too confronting	40	16%
I prefer to deal with it on my own	39	16%
Embarrassed to talk with someone about my problems	38	16%
Not able to get to a therapist	38	16%
I didn't think it would help	33	13%
Lack of time	32	13%
I don't really understand what therapy involves	25	10%
It would hurt my reputation or career	17	7%
I recovered	15	6%
Someone told me not to do it	3	1%

### 4. Treatment: Internet Sample

#### Would you try Internet therapy?

One hundred and eighty-one (74%) of the Internet sample indicated they *would possibly try* or *would definitely try* Internet therapy for PTSD, compared with 12 (5%) who reported that they *would possibly not try* or *would definitely not try* Internet therapy. The remaining 47 (19%) had a neutral preference.

#### Preference for Internet therapy over face-to-face

Seventy one (29%) of the Internet sample reported that they would *mildly, strongly prefer* or *very definitely prefer* Internet therapy compared with 78 (32%) indicating that they would *mildly, strongly prefe*r or *very definitely prefer* face-to-face treatment, while the remaining 95 (39%) reported no preference.

## Discussion

The primary aim of the present study was to identify characteristics of Australian adults with PTSD who completed an Internet-based survey, and to compare their characteristics with those identified in a national survey. The secondary aims were to examine evidence-based service utilization and barriers to treatment, as well as the acceptability of Internet therapy to the Internet sample. This knowledge allows us to better understand the key similarities and differences between Internet samples and the general population, as well as providing information about the reasons why individuals seek Internet-based treatment. All participants met diagnostic criteria for PTSD or were likely to meet such criteria based on a psychometrically valid cut-off on a measure of PTSD symptom severity.

### Comparison of Demographic Characteristics and Symptom Severity

The Internet sample reported higher levels of education than the national sample; however, they did not differ in age, gender, marital status or employment. Overall, it appears that the Internet sample is generally representative of the wider population of those with PTSD.

Importantly, the Internet sample had considerably higher levels of psychological distress and disability than the national sample. The magnitude of these symptoms are similar to those reported in a recent comparison of an Internet treatment sample with a national sample [25]. The severity of symptoms of PTSD in the Internet sample in the present study as measured on the PCL-C (mean = 60.4, SD = 13.1) was as large as that reported in a recent treatment trial of Internet therapy for PTSD (mean = 57.7, SD = 12.7) [19], [34] and at least as severe as reported in a face-to-face outpatient treatment program for PTSD in civilians (mean = 48.8, SD = 9.2) [35]. Moreover, 71% of the Internet sample reported experiencing four or more types of trauma over their lifetime. This latter finding is consistent with the prevalence rate of multiple trauma episodes in other studies, which report that up to 77% of diagnosed PTSD sufferers experience multiple trauma types [36]. Multiple types of trauma are associated with higher symptom levels [37], which is also consistent with the pattern observed here. These results indicate that people exploring treatment options via the Internet have non-trivial symptoms.

### Service Utilization and Barriers to Treatment

Less than half of the Internet sample reported receiving treatment for PTSD despite having significant symptoms of PTSD. For those who did receive treatment, evidence-based approaches appear to be utilized, with 68% of the Internet sample reporting receiving treatment using CBT and 79% reporting having been treated for PTSD using a pharmacological approach. This is inconsistent with US and Australian data which indicate that uptake of evidence-based interventions for psychiatric conditions is low [1], [38], [39]. Many of those in the Internet sample (48%) were unsure about the availability of local services and 15% believed that there was no one in their area who could provide treatment for PTSD. We are not able to determine whether these perceptions are correct, but they may partially account for the finding that 49% of the Internet sample reported not having received treatment for PTSD. In addition to the perceptions of limited accessibility to face-to-face treatment, the Internet sample indicated that other barriers to receiving treatment included the financial costs associated with treatment (43%), previous poor treatment response (30%), not wanting to take medication (24%), and not perceiving personal distress as severe enough to warrant treatment (21%), the latter being a frequently reported barrier to treatment seeking amongst trauma survivors [28].

### Treatment Preferences and Acceptability of Internet Treatment

A large proportion of Internet survey respondents (74%) reported they would be prepared to try Internet therapy for PTSD. Consistent with this, approximately one third of respondents reported a preference for face-to-face treatment, another third for Internet therapy, and a final third reported no preference. This is consistent with other recent studies indicating consumers are prepared to try Internet treatment for anxiety and depression [40], [41], although to our knowledge, this is the first study to enquire specifically from people with PTSD. Importantly, several respondents also indicated in text responses that they were interested in Internet therapy as an adjunct to their current face-to-face treatment. We conclude that Internet therapy is an acceptable choice for consumers with PTSD, and that such an approach may be potentially attractive as an adjunct or alternative to the traditional face-to-face service model.

### Limitations

One limitation of this study was the absence of a clinical diagnosis of PTSD in the Internet sample. This was mitigated by use of a recommended clinical cut-off on the PCL-C along with screening questions to exclude Acute Stress Disorder and to confirm the experience of a traumatic event. Additional limitations included the use of several non-validated measures, although these were based on those used in similar studies [1], [40]. The treatment definitions provided may not have been sufficiently explanatory to prevent misunderstandings and the validity of self-reports about service utilization was not checked, potentially biasing the rates of evidence-based service utilization. Finally, the Internet sample were visitors to a website involved in conducting online clinical trials, and it is likely that they had a pre-existing favourable bias towards Internet treatment.

### Conclusions and Future Research

The results of the current study indicate Australian respondents to an Internet survey with symptoms of PTSD were similar to a broader sample of Australians with PTSD. Approximately 50% of respondents denied previously receiving treatment despite experiencing clinically significant symptoms of PTSD. Reported barriers to treatment included the perception of limited face-to-face treatment options, costs of treatment, lack of response to prior treatment, unwillingness to use medication and not perceiving personal distress as severe enough to warrant treatment. Importantly, of those who reported receiving treatment, at least 68% reported receiving an evidence-based treatment. These data suggest this population of people with PTSD may be accessing appropriate care; however, almost half the Internet sample had not previously received treatment for PTSD. Most survey respondents indicated they were willing to try Internet treatment.

These data provide preliminary evidence for demand and acceptability of iCBT programs for PTSD. Emerging evidence indicates such treatments can be effective [15], [16], [18], [19], [20], [42], indicating the potential of Internet treatment as a strategy for improving access to evidence-based care for people with PTSD.

## Supporting Information

Appendix S1 Treatment Definitions Used in Survey.
(DOC)
