# Supplementary material for: Characteristics and Treatment Preferences of People with Symptoms of Posttraumatic Stress Disorder: An Internet Survey
Source: PLoS One. 2011 Jul 19;6(7):e21864. doi: 10.1371/journal.pone.0021864 (PMC3139581; doi:10.1371/journal.pone.0021864)
Supplement: Appendix S1 — Treatment Definitions Used in Survey. (DOC) [file pone.0021864.s001.doc]

**Appendix.** Treatment Definitions Used in Survey.

| **Treatment** | **Definition Provided in Survey** |
| --- | --- |
| Information | *Information about mental illness, its treatment, and available services* |
| Medication | *Medicine or tablets for PTSD symptoms* |
| Internet Therapy | *Internet therapy (therapy delivered via the Internet)* |
| Psychotherapy | *Psychotherapy – discussion about the causes of your symptoms that stem from your childhood and/or where the therapist interprets the meaning of what you do to help you gain insight* |
| Cognitive Behavioral Therapy (CBT) | *Cognitive behaviour therapy (CBT) – learning how to change your thoughts, behaviours and emotions and confronting feared situations in a gradual way (such as via exposure to triggers or re-evaluating specific beliefs)*. |
| Counselling | *Counselling – help to talk through your day to day problems* |
